# On the size of tokamak fusion power plants

**DOI:** 10.1098/rsta.2017.0437

**Published:** 2019-02-04

**Authors:** Hartmut Zohm

**Affiliations:** Max-Planck-Institut für Plasmaphysik, Boltzmannstr. 2, 85748 Garching, Germany

**Keywords:** tokamak, fusion power plant, exhaust, confinement, stability limits

## Abstract

Figures of merit for future tokamak fusion power plants (FPPs) are presented. It is argued that extrapolation from present-day experiments to proposed FPPs must follow a consistent development path, demonstrating the largest required leaps in intermediate devices to allow safe extrapolation to an FPP. This concerns both plasma physics and technology. At constant plasma parameters, the figures of merit depend on both major radius *R* and magnetic field *B*. We propose to use the term ‘size’ for a combination of *R* and *B* to avoid ambiguities in scaling arguments. Two routes to FPPs are discussed: the more conventional one increasing *R*, based on the assumption that *B* is limited by present technology; and an alternative approach assuming the availability of new technology for superconducting coils, allowing higher *B*. It is shown that the latter will lead to more compact devices, and, assuming a criterion based on divertor impurity concentration, is in addition more favourable concerning the exhaust problem. However, in order to obtain attractive steady-state tokamak FPPs, the required plasma parameters still require considerable progress with respect to present experiments. A credible strategy to arrive at these must hence be shown for both paths. In addition, the high-field path needs a demonstration of the critical technology items early on.

This article is part of a discussion meeting issue ‘Fusion energy using tokamaks: can development be accelerated?’.

## 1. Introduction

The development of nuclear fusion as an energy source using magnetic confinement in toroidal geometry has progressed along a path on which devices progressively became larger in terms of the torus' major radius *R* and also made use of increased strength of the confining magnetic field *B* in order to reach conditions close to ‘ignition’, i.e. where the energy loss from the plasma by conduction and convection is compensated by the heating of the plasma through the α-particles generated in the fusion reaction^1^ [1]. The ignition criterion can be expressed by the so-called triple product *nTτ*_E_, where *n* is the plasma density, *T* the ion temperature and *τ*_E_ the energy confinement time, i.e. ratio of plasma stored energy to the loss power; *τ*_E_ has been found to strongly increase with both *R* and *B*. Today, the largest tokamak JET (*R* = 3 m, *B* = 4 T) can achieve conditions close to breakeven (where the fusion power generated is as large as the power deposited in the plasma to sustain it), and the next-step device ITER (*R* = 6.2 m, *B* = 5.2 T), presently under construction, aims at increasing this ratio to 10. However, an economically attractive fusion power plant (FPP) will require even larger values and has to fulfil several other conditions, such as long pulses or steady-state operation (effectively allowing continuous electricity production).

In order to determine the parameters of a future tokamak FPP, an extrapolation is hence used, based on a combination of experimental results and theoretical understanding. Assuming a certain plasma performance in terms of energy confinement, stability and exhaust, one can determine the required machine parameters such as *R*, *B* and the required auxiliary heating power *P*_AUX_ or the current drive (CD) power *P*_CD_ to drive the plasma current. These have to be consistent with the assumptions about technology, e.g. the value of *B* is usually limited by the critical current in the superconductor or by the forces on the inner leg of the toroidal field coils.

A ‘roadmap’ to a tokamak FPP usually involves several steps that develop the necessary physics and technology to arrive at an attractive FPP. For example, the EU Roadmap [2] builds on a demonstration of dominant self-heating of the plasma by α-particles in ITER, followed by the demonstration of a closed fuel cycle in DEMO. A credible strategy must be presented for ‘safe’ extrapolation within a roadmap to minimize the risk of failure in a step. In the EU Roadmap, the present assumption is that the physics necessary for the plasma scenario will essentially be demonstrated in ITER, and DEMO is no longer considered an experimental device. Likewise, the present EU DEMO design builds on ITER technology where possible.

In this paper, we will examine different extrapolation paths to a tokamak FPP, and argue that the term ‘size’ should comprise both geometrical size *R* as well as magnetic field strength *B*. In the next section, we propose four figures of merit that have to be optimized to arrive at an efficient FPP. These will be formulated using a simple zero-dimensional (0D) model that captures the dependence on the essential plasma physics parameters as well as on the machine parameters *R* and *B*. In the following section, we discuss a route to an FPP based on increasing *R*, assuming that the value of *B* is limited by present-day technology. Then, a route that increases *B*, assuming progress with superconductor technology, is discussed. In the final section, conclusions are drawn for both development lines.

## 2. Figures of merit for tokamak fusion power plants

In this section, we define figures of merit for tokamak FPPs and show how to derive them from a simple 0D model that involves the basic plasma parameters as well as the size parameters *R* and *B*. The choice of plasma parameters is similar to the model presented in [3] and [4], and for the derivation of the equations, we refer the reader to the detailed descriptions given there. We use the following 0D plasma parameters:

— Normalized pressure *β*_N_ = *β*/(*AI*_p_/(*RB*)), where *β* = 〈*p*〉/(*B*^2^/(2*μ*_0_)) is the ratio of average kinetic pressure to magnetic field pressure, *A* is the torus' aspect ratio, i.e. the ratio of major to minor radius, and *I*_p_ is the plasma current. The quantity *β*_N_ is limited by ideal magnetohydrodynamic (MHD) stability. Since, under optimum conditions, the fusion power *P*_fus_ increases quadratically with *β*, this so-called *β*-limit effectively limits the fusion power achievable for given *B*.
— Confinement relative to the ITER confinement scaling: it is envisaged that ITER will operate under ‘H-mode’ plasma conditions, where a transport barrier at the plasma edge increases *τ*_E_ with respect to (w.r.t.) so-called L-mode conditions [1]. The confinement quality is hence measured by *H* = *τ*_E_/*τ*_E,ITER98_ where *τ*_ITER98_ is the energy confinement time predicted by the empirical ITER H-mode scaling [5]. Assuming *H* > 1 hence means that one assumes that confinement is better than the value predicted by the ITER scaling law.
— Electron density relative to the empirical ‘Greenwald’ density limit *f*_GW_ = *n*_ave_/*n*_GW_ with *n*_GW_ = *I*_p_/(*πA*^2^/*R*^2^) [6]. Here, *n*_ave_ is the line-averaged electron density in units of 10^20^ m^−3^. Since fusion power scales quadratically with *n*, the density limit also limits the fusion power achievable for given plasma current *I*_p_.
— Safety factor *q* = *Sq*_cyl_ where *q*_cyl_ = 5 *μ*_0_ (*R*/*A*^2^)(*B*/*I*_p_) is the cylindrical safety factor and *S* is the so-called shape factor that takes into account the effect of toroidicity and shaping of the plasma cross section. In the present study, *S* has been held constant and hence the shape dependence of other quantities has been neglected. The safety factor is limited by the ideal MHD kink limit, effectively limiting the maximum achievable plasma current, which in turn limits the density by the Greenwald limit. Also, *τ*_E_ increases roughly linearly with *I*_p_ so that limiting *I*_p_ will also limit *τ*_E_.
— Power flux across the separatrix relative to the H–L threshold *f*_LH_ = *P*_sep_/*P*_HL_, where *P*_sep_ is the power crossing the separatrix in charged particles, i.e. radiation is not included. A minimum power *P*_sep_ is needed to transit to H-mode confinement conditions, and for this threshold value *P*_LH_, the empirical scaling [7] is used. Once H-mode conditions are established, the back transition to L-mode conditions occurs when *P*_sep_ falls below a threshold value *P*_HL_. In our study, we assume *P*_HL_ = *P*_LH_, neglecting a possible favourable hysteresis *P*_HL_ < *P*_LH_. This assumes implicitly that radiation losses are controlled, e.g. by addition of suitable impurities, such that the desired *f*_LH_ is reached. For an extension using the simple model presented here, taking into account the effect of impurity seeding on confinement, see [8].

The figures of merit used in our analysis are the following:

— Fusion power *P*_fus_: this quantity is roughly equal to the thermal power generated by the plant. The self-heating of the plasma through fusion-produced α-particles is just *P*_fus_/5:
  2.1 $$P_{\mathrm{fus}}=c_{\mathrm{fus}}\frac{\beta_{N}^{2}B^{4}R^{3}}{q^{2}A^{4}}.$$
— Ratio of fusion power to auxiliary power needed to sustain the plasma from power balance, *Q*_PB_:
  2.2 $$Q_{\mathrm{PB}}=\frac{P_{\mathrm{fus}}}{P_{\mathrm{AUX}}}=\frac{1}{c_{\mathrm{PB}}(q^{3.1}A^{3.53}/(H^{3.23}\beta_{N}^{0.1}R^{2.7}B^{3.7}))-1/5}.$$
— Note the ‘resonance denominator’, as the α-heating fully sustains the plasma (ignited plasma). Here *Q*_PB_ should be minimized to minimize the recirculating electrical power in the plant which, according to [8], is inversely proportional to *Q*_PB_ in the limit that *P*_AUX_ dominates the auxiliary electrical power needed to run the plant.
— Ratio of fusion power to CD power needed to sustain the plasma current, *Q*_CD_. Like *Q*_PB_, this quantity should be minimized to minimize recirculating power. In this paper, we assume that the tokamak operates in steady state, and we use a generic scaling of the CD efficiency with *T/n*, where *T* is the plasma temperature. This leads to
  2.3 $$Q_{\mathrm{CD}}=\frac{P_{\mathrm{fus}}}{P_{\mathrm{CD}}}=c_{\mathrm{CD}}\frac{\beta_{N}^{3}R^{3}B^{3}}{A^{3}f_{\mathrm{GW}}^{2}(5+Z_{\mathrm{eff}})(1-c_{\mathrm{BS}}\sqrt{A}\;q\beta_{N})}.$$
— Note that also this quantity exhibits a ‘resonance denominator’ when the so-called bootstrap current fraction *I*_BS_*I*_P_ ∼ *A*^1/2^*qβ*_N_ attains 100%. The bootstrap current is a self-generated thermo-electrical toroidal current with its magnitude proportional to the radial gradient of the pressure gradient. For a fully consistent solution, *Q*_CD_ = *Q*_PB_, but as the two quantities assume large values, their difference will not influence the power balance strongly and hence this condition is not always enforced in this paper. As outlined in [3], equations (2.1), (2.2) and (2.3) assume that the temperature range is close to that of the optimum reactivity, i.e. around 10–20 keV average temperature, which must be verified afterwards on inserting the assumed density *n* = *f*_GW_*n*_GW_.
— Concentration of impurities in the divertor, *f_Z,_*_div_: the power flowing across the separatrix is guided by the magnetic field to the first wall in a very narrow region, potentially leading to high local heat loads. Here, we assume that the power across the separatrix is so large that impurities have to be introduced into the region outside the separatrix to lead to additional dissipation. We use a criterion derived by Reinke [9]
  2.4 $$f_{Z,\mathrm{div}}=c_{\mathrm{exh}}\frac{f_{\mathrm{LH}}^{1.14}q^{0.32}B^{0.88}R^{1.33}}{f_{\mathrm{GW}}^{1.18}}.$$
— This criterion takes into account recent results that indicate that the width of the power-conducting layer outside the separatrix scales with the poloidal gyro-radius [10]. In our parameter studies, we will assume that *f_Z,_*_div_ takes on the value foreseen for the ITER *Q* = 10 scenario, meaning that the exhaust scenario would be demonstrated in ITER.

Equations (2.1)–(2.4) allow one to calculate the figures of merit for particular choices of the plasma parameters as well as *R* and *B*. Since we are interested in scaling with these parameters, we will plot, for particular choices of the plasma parameters, lines of constant figures of merit in *R–B* space to understand the interdependences.

**Figure 1.**
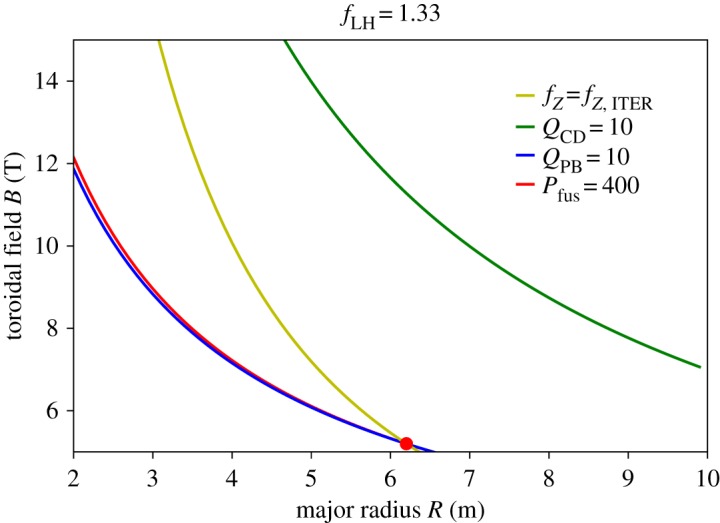
Lines of constant figures of merit in *R–B* space for the ITER *Q* = 10 scenario (*A *= 3.1, *q *= 3.1, *H *= 1, *β*_N_ = 1.8, *f*_GW_ = 0.85, f_LH_ = 1.33). The red point shows the ITER design point.

As an example, we show in figure 1 the figures of merit for the ITER *Q* = 10 scenario (*A *= 3.1, *q *= 3.1, *H *= 1, *β*_N_ = 1.8, *f*_GW_ = 0.85, *f*_LH_ = 1.33), where the figures of merit have been set to the ITER values *P*_fus_ = 400 MW, *Q*_PB_ = 10 and *f*_Z_ = *f*_*Z*,ITER_ (the particular value has not been calculated explicitly and will only be used in a relative way).

It can be seen that the lines for the three figures of merit cross in the ITER point, which fulfils all three criteria. Also plotted in figure 1 is the condition *Q*_PB_ = 10, which is not fulfilled by far. This is not surprising because the ITER *Q* = 10 scenario is aimed at pulsed operation, but it also shows that, with this optimization strategy, it will not be easy to obtain steady state.

It can also be seen in figure 1 that the lines for *Q*_PB_ and *P*_fus_ are almost on top of each other in the whole parameter range. This is due to the very similar scaling of the two figures of merit, which means as one is fixed, the other one will not vary significantly along this path in the *R–B* plane. This has been interpreted as ‘*P*_fus_ and *Q*_PB_ not depending on size’ [11,12], but, as can be seen from, for example, fig. 2 in [11], *B* varies throughout the whole scan and the same *P*_fus_ and *Q*_PB_ at smaller size imply a higher *B*. For the purpose of the present study, we propose the ‘size’ of a tokamak should be measured by a combination of *R* and *B*, for example, *BR^3/4^* if *Q*_PB_ and *P*_fus_ are the figures of merit considered. We note, however, that there is no unique combination of the two parameters to describe all four figures of merit described above, and for *Q*_PB_, it will depend on the exponents of the (empirical) confinement scaling law used. For the remainder of the paper, we will hence examine the figures of merit in *R–B* space, similar to figure 1. Note that for a fixed set of the figures of merit (*P*_fus_, *Q*_PB_, *Q*_CD_*, f_Z_*), the acceptable area in *R–B* space is above the lines for the first three parameters (they should be maximized) and below the line of constant *f_Z_*. This will define an acceptable area in *R–B* space which, in some cases, may not exist (such as in figure 1 if *Q*_CD_ ≥ 10 was taken as a requirement).

We will now examine two different strategies to increase the ‘size’ towards an FPP, namely increasing *R* at roughly constant *B* (assuming a technology limit on *B*) and increasing *B* while at the same time decreasing *R* (assuming that new magnet technology exists).

## 3. The road to a tokamak fusion power plant: increasing geometric size

**Figure 2.**
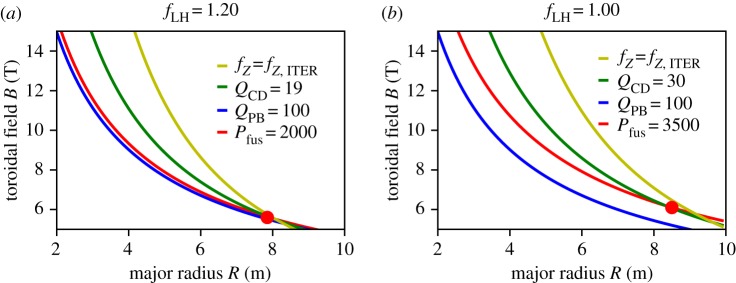
Lines of constant figures of merit in *R–B* space for the stepladder scenario (*A *= 3.1, *q *= 4.5, *H *= 1.2, *β*_N_ = 3.5, *f*_GW_ = 1.2). The red point shows the DEMO (*a*) and the FPP (*b*) design points.

In this section, we discuss the ‘conventional’ approach of scaling up tokamaks in order to reach parameters necessary for economic production of power. As an example, we look at the recently developed ‘stepladder’ strategy that is based on the assumption of steady-state tokamak operation in a ‘hybrid H-mode’ regime, i.e. higher *q* and *β*_N_ than in ITER baseline operation, which according to (2.3) will increase *Q*_CD_. At the same time, the higher *q* means a reduction in *Q*_PB_, which is compensated by choosing a regime in which it is expected that *τ*_E_ exceeds that of ITER baseline operation, i.e. *H* = 1.2. The ITER research plan actually foresees a phase in which, after demonstrating its *Q* = 10 goal, such a steady-state scenario will be developed and the parameters chosen in the stepladder approach are compatible with this plan. In the ‘stepladder’ approach, an FPP design point is chosen that sits at the upper limit of *B*, assuming conventional superconducting magnet technology (*B* = 6.1 T on the plasma axis) and just fulfils the requirement of acceptable recirculating power (*Q*_PB_ = 30, corresponding to less than 20% of recirculating power). DEMO is then scaled down in order to fulfil the EU stakeholder requirement of 500 MW net electrical power. We note here that, as discussed in detail in [4], the simple equations (2.1)–(2.3) slightly underpredict the size of the machines because the temperature is already exceeding the optimum value. Hence, 1.5-dimensional (1.5D) transport modelling has been employed in [4] to confirm the basic findings of the 0D model. The *R*–*B* diagrams for DEMO and FPP in this strategy are shown in figure 2.

Both design points fulfil the imposed requirements on *Q*_CD_ and *P*_fus_, while actually *Q*_PB_ exceeds substantially *Q*_CD_, indicating that the power requirements are indeed no longer set by the power balance but rather by the steady-state constraint. Concerning exhaust, it can be seen that both points lie below the ITER yellow similarity line, but this was achieved by reducing the margin w.r.t. the LH threshold power, i.e. *f*_LH_ was reduced from 1.33 to 1.2 (DEMO) and further down to 1.0 for the FPP. This means that, assuming the ITER exhaust solution will be used for DEMO and FPP, FPP is right at the margin of the approach. It is interesting to note that criterion (2.4) is fulfilled although the exhaust criterion used in [4] was actually limiting the value of *P*_sep_, the heat flux into the scrape-off-layer volume, to the ITER value, which, assuming the Eich scaling for the width [10], can be written as *P*_sep_*B*/(*qAR*) = const. This is a consequence of the stepladder strategy keeping the absolute value of the density constant (precisely to deal with the exhaust) and hence *B*/(*qR*) = const. In this case, *f*_LH_ ∼ 1/*R*^2.8^ on the stepladder, while *f*_LH_ ∼ 1/*R*^2^ in (2.4) if all other parameters are kept constant. Hence, for the limited range of extrapolation, the difference between the two criteria is not too large. For larger variations (as will be discussed in the next section), the difference could be substantial.

In summary, a strategy in which the magnetic field is limited to that of present superconducting magnet technology, i.e. of the order of 6 T on axis for *A* = 3, will lead to devices of the order of *R* = 8–9 m. In order to satisfy the exhaust criterion (2.4), *f*_LH_ is usually close to 1 and *f*_GW_ is chosen at the upper limit, together with *q* not being too large. At FPP size, this is also needed to limit the ion temperature, which is usually already above the optimum value due to the lowish density due to the unfavourable scaling of the Greenwald density (*n*_GW_* ∼ B*/(*qR*)). The latter two choices (highish *f*_GW_ and lowish *q*) on the other hand imply a limited *Q*_CD_ as can be seen from (2.3). These findings from the simple 0D model are fully in line with a more extensive analysis using a 1.5D transport code in conjunction with a divertor model [13].

## 4. The road to a tokamak fusion power plant: increasing magnetic field

From the discussion following equations (2.1)–(2.4), it is clear that extrapolation to reactor-like conditions can also be done by increasing the magnetic field. This is based on the assumption that, in future, magnet technology will be available to exceed the values typical for present large-scale superconducting fusion magnets. The present technology, as is used, for example, in ITER (NbTi or Nb_3_Sn), is limited by the maximum value of *B* that occurs at the inner leg of the toroidal field coil. The above-mentioned conventional limit of 6 T on axis corresponds to roughly 12–13 T at the inner leg, the value being set by the critical current density in the superconductor. With the prospects of high-temperature superconductors of the REBCO (rare earth barium copper oxide) type becoming available for use in fusion magnets, it is expected that this limit could be increased to around 20 T, with the limitation now coming from the need to support the increased mechanical stresses (e.g. [14]). Assuming that a satisfactory engineering solution can be found, we now analyse the possibilities that an increase of the central magnetic field up to a value of around 10 T can offer.

**Figure 3.**
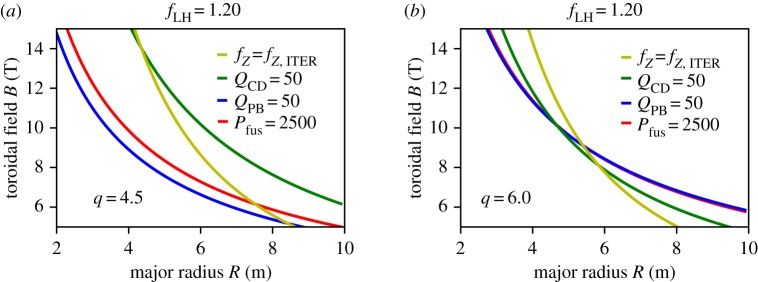
Lines of constant figures of merit in *R–B* space for the stepladder scenario (*A *= 3.1, *H *= 1.2, *β*_N_ = 3.5, *f*_GW_ = 1.2). At *q* = 4.5 (*a*), it is not possible to find a solution at high *Q*_CD_ = 50 up to *B* = 15 T, but increasing *q* to 6 (*b*) opens up a parameter space above 9.5 T for such a solution.

Starting from the stepladder approach shown in figure 2, it can be seen that a region below the yellow exhaust line, but above the other lines opens up as one moves to larger *B* and smaller *R*. In figure 3, we analyse how *Q*_CD_ could be increased by moving into this region, while at the same time satisfying the exhaust criterion. Figure 3*a* shows the *R–B* space for the same plasma physics parameters as used in the stepladder DEMO in figure 2*a*, but now with a slightly increased fusion power and the figure of merit *Q*_CD_ increased to 50 (which would correspond to a very attractive recirculating electrical power of less than 10%). It is clear that the operational space for this regime would only open up above 15 T, way beyond our assumptions even for improved magnet technology. However, because exhaust is more relaxed at higher *B*, lower *R*, we can now increase *q*, which has a stronger effect on *Q*_CD_ than on *f_Z_*_,ITER_, at least if *β*_N_ is high enough. In figure 3*b*, this is demonstrated by increasing *q* to 6, at otherwise unchanged parameters. Now, an operational space opens up that would allow one to locate a point at e.g. *R* = 5 m and *B* = 10 T that would actually exceed the figures of merit *Q*_CD_ and *f_Z_*_,ITER_ for the FPP shown in figure 2, at strongly reduced size. Thus, we conclude that it is possible to find more compact solutions that fulfil all requirements on the figures of merit at higher *B*. We also conclude that, with the physics assumptions made for the EU stepladder, an attractive FPP could be placed at *R* = 5 m, *B* = 10 T.

**Figure 4.**
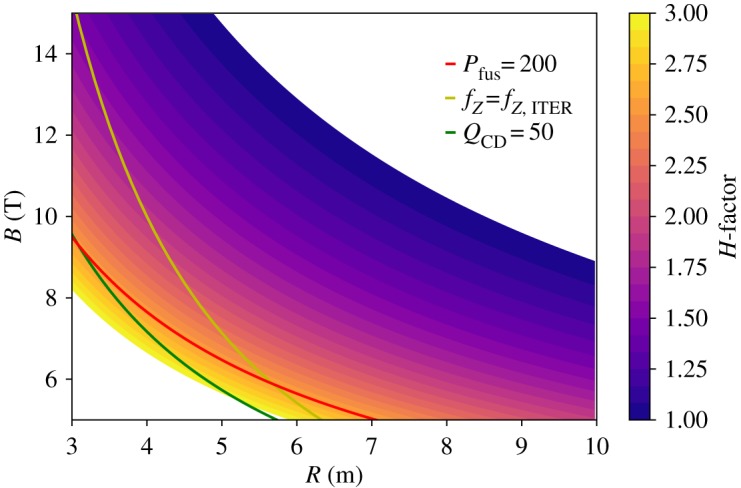
Lines of constant figures of merit in *R–B* space for relaxed physics assumptions w.r.t. the stepladder scenario (*A *= 3.1, *β*_N_ = 3.0, *f*_GW_ = 1.0). For the operational space opening up at low *R*, high *B*, the *H*-factor, shown as contours, has to be quite high.

Figure 3 indicates a possible approach to benefit from the future availability of higher-field fusion magnets. The question remains if other strategies can lead to even more compact devices or if the plasma performance requirements can be relaxed w.r.t. to the stepladder parameters used in figure 3. One parameter that could still be used for optimization is the aspect ratio *A*, which, taken to its extreme, would lead to the spherical torus approach, as advocated, for example, in [15]. In a spherical torus, *A* approaches 1, while conventional tokamaks have an aspect ratio around 3. It is at present not clear if the confinement and stability parameters of spherical tori follow the same scaling laws as those of conventional aspect-ratio tokamaks, and hence we do not study this approach here, but rather refer to [15]. Instead, we stay with a conventional aspect ratio of *A* = 3.1. In order to find a solution with reduced plasma performance, we decrease *β*_N_ to 3.0 and *f*_GW_ to 1, keeping *f*_LH_ = 1.2. Following the same strategy as above, we increase further *q* to increase the bootstrap fraction such that *Q*_CD_ can still have a high value. At *q* = 8.2, we can obtain an operational window^2^ at low *R*, down to *R* = 3 m at *B* = 10 T. This is shown in figure 4. However, we note that now the fusion power is as low as 200 MW and the *H*-factor needed to guarantee at least *Q*_PB_ = Q_CD_, as indicated by the coloured contour levels in figure 4, must be postulated to substantially exceed the ITER level (*H* ≥ 2 in the region of interest). This is of course due to the high *q* which reduces confinement, which has to be compensated by high *H*-factor.

Thus, we conclude that it is not possible to access the region at *R* < 5 m unless more optimistic assumptions about the physics performance are made. This is actually consistent with the ARC [14] study that assumed an *H*-factor of 1.8 and more aggressive shaping and temperature profiles than used in our study to increase at the same time the fusion power.

## 5. Discussion and conclusion

In this contribution, we have analysed the size of future tokamak power plants. We have shown that the term ‘size’ involves a combination of the geometrical size and the strength of the confining magnetic field. For our figures of merit, however, no unique combination of *R* and *B* can be given that would describe ‘size’ in one parameter. This has been shown to be different if only core plasma physics is described, because the usual dimensionless parameters *β*, ρ* and ν* can be uniquely described in so-called dimensionless engineering parameters *R*^5/4^*B* and *PR*^3/4^ [16]. We have used a different approach here, mainly because both the empirical Greenwald limit and the exhaust figure of merit cannot be expressed in these dimensionless variables, which, for the latter, can be easily understood because atomic physics plays a role which sets an absolute energy scale.

Different strategies to increase the ‘size’ of tokamaks to become viable FPPs have been discussed. Increasing geometrical size, i.e. major radius *R*, and limiting *B* to values that can be achieved using present-day superconductors, typical power plants are of the size range *R* = 8–9 m, limited by the ability to exhaust the energy carried across the separatrix in charged particles through the divertor. In addition, aiming at steady-state tokamaks, the fusion gain *Q* is usually limited by the power requirement for CD. Assuming that magnet technology that allows higher values of *B* will be available in future, e.g. by using REBCO tapes that have a higher critical current density, it would be possible to decrease *R* at otherwise constant plasma performance. An operational point around 5 m, 10 T has been pointed out, at which a tokamak could produce 2.5 GW of fusion power in steady-state conditions, using plasma performance parameters that lie within the range of present tokamak experiments. Decreasing the size further needs an improvement in confinement and/or stability beyond these parameters, more typical of what is achieved in present-day experiments under transient conditions only. Hence, this route will need substantial development in both plasma physics and magnet technology.

Concerning typical roadmaps to fusion power using tokamaks, it will be important to demonstrate such improved parameters, in both physics and technology, as soon as possible. If major development steps are still to be demonstrated in a nuclear device, the risk for extrapolation will be enhanced considerably because these are less flexible for experimentation. This element must be taken into account when evaluating ‘fast’ strategies that aim at an accelerated development of fusion energy using tokamaks.

## Footnotes

1 We assume that the DT fusion reaction is used, where D + T → He + n + 17.6 MeV.

2 Note that the precise value of *q* becomes critical in this region where the bootstrap fraction is high, indicating that a more sophisticated analysis will be required to confirm the exact result.

## Data accessibility

This article has no additional data.
